# Supplementary material for: Fitness effects of altering gene expression noise in Saccharomyces cerevisiae
Source: eLife. 2018 Aug 20;7:e37272. doi: 10.7554/eLife.37272 (PMC6133559; doi:10.7554/eLife.37272)
Supplement: Transparent reporting form [file elife-37272-transrepform.docx]

***eLife’s* transparent reporting form**

We encourage authors to provide detailed information *within their submission* to facilitate the interpretation and replication of experiments. Authors can upload supporting documentation to indicate the use of appropriate reporting guidelines for health-related research (see [EQUATOR Network](http://www.equator-network.org/%20)), life science research (see the [BioSharing Information Resource](https://biosharing.org/)), or the [ARRIVE guidelines](http://www.plosbiology.org/article/info:doi/10.1371/journal.pbio.1000412) for reporting work involving animal research. Where applicable, authors should refer to any relevant reporting standards documents in this form.

If you have any questions, please consult our Journal Policies and/or contact us: [editorial@elifesciences.org](mailto:editorial@elifesciences.org).

**Sample-size estimation**

- You should state whether an appropriate sample size was computed when the study was being designed
- You should state the statistical method of sample size computation and any required assumptions
- If no explicit power analysis was used, you should describe how you decided what sample (replicate) size (number) to use

Please outline where this information can be found within the submission (e.g., sections or figure legends), or explain why this information doesn’t apply to your submission:

The choice and construction of strains analyzed to characterize empirical expression levels is described in methods (pages 12-16) and results/discussion (pages 4-5). The choice and construction of strains analyzed to characterize fitness is described in methods (pages 16-19) and results/discussion (pages 5-6).

Power analyses were performed by simulation to estimate the appropriate choice of experimental conditions (including sample size) to allow accurate and sensitive detection of fitness differences. These simulations are described in the “Fitness” section of the methods (pages 23-24). For expression assays, sample size was decided based on our previous experience using the same fluorescent reporter and the same flow cytometer (see Gruber *et al*. 2012; Duveau *et al.* 2014; Metzger *et al*. 2015; Metzger *et al.* 2016; Duveau *et al.* 2017a; Duveau *et al.* 2017b).

**Replicates**

- You should report how often each experiment was performed
- You should include a definition of biological versus technical replication
- The data obtained should be provided and sufficient information should be provided to indicate the number of independent biological and/or technical replicates
- If you encountered any outliers, you should describe how these were handled
- Criteria for exclusion/inclusion of data should be clearly stated
- High-throughput sequence data should be uploaded before submission, with a private link for reviewers provided (these are available from both GEO and ArrayExpress)

Please outline where this information can be found within the submission (e.g., sections or figure legends), or explain why this information doesn’t apply to your submission:

Numbers of replicate experiments performed are described in each figure legend and Source Data file as well as in the methods (pages 12-19) and results sections (pages 4-6). As described in the methods (pages 12-19), replicates represent independent cultures and/or competitions of the same starting genotype(s). Expression and fitness data for all replicates of all genotypes are available in Supplementary File 1.

When possible, independent clones representing the same mutation were included as additional independent samples. These cases are detailed in Supplementary File 1, the methods (page 16), and Figure 2 - figure supplement 2C&D.

As described in the methods (page 26, 27) and in R scripts (Supplementary File 3), outliers were defined as those samples that fell outside five times the median absolute deviation of all replicates for the same genotypes.

All primers used to generate transformants, perform pyrosequencing and confirm genotypes by Sanger sequencing are listed in Supplementary File 2.

**Statistical reporting**

- Statistical analysis methods should be described and justified
- Raw data should be presented in figures whenever informative to do so (typically when N per group is less than 10)
- For each experiment, you should identify the statistical tests used, exact values of N, definitions of center, methods of multiple test correction, and dispersion and precision measures (e.g., mean, median, SD, SEM, confidence intervals; and, for the major substantive results, a measure of effect size (e.g., Pearson's r, Cohen's d)
- Report exact p-values wherever possible alongside the summary statistics and 95% confidence intervals. These should be reported for all key questions and not only when the p-value is less than 0.05.

Please outline where this information can be found within the submission (e.g., sections or figure legends), or explain why this information doesn’t apply to your submission:

Statistical analyses are explained in detail in the methods (pages 19 – 30). R scripts used to perform statistical analyses are available in Supplementary File 1. Statistical results are shown in the results section and on the relevant figures.

(For large datasets, or papers with a very large number of statistical tests, you may upload a single table file with tests, Ns, etc., with reference to sections in the manuscript.)

**Group allocation**

- Indicate how samples were allocated into experimental groups (in the case of clinical studies, please specify allocation to treatment method); if randomization was used, please also state if restricted randomization was applied
- Indicate if masking was used during group allocation, data collection and/or data analysis

Please outline where this information can be found within the submission (e.g., sections or figure legends), or explain why this information doesn’t apply to your submission:

Not applicable to this submission.

**Additional data files (“source data”)**

- We encourage you to upload relevant additional data files, such as numerical data that are represented as a graph in a figure, or as a summary table
- Where provided, these should be in the most useful format, and they can be uploaded as “Source data” files linked to a main figure or table
- Include model definition files including the full list of parameters used
- Include code used for data analysis (e.g., R, MatLab)
- Avoid stating that data files are “available upon request”

Please indicate the figures or tables for which source data files have been provided:

Numerical data used to produce the following figures were provided in Source Data Files: Figure 1B-D, Figure 2C, Figure 3, Figure 4, Figure 6B & 6D, Figure 2 – figure supplement 1, Figure 5 – figure supplement 1. The correspondence between Source Data Files and Figures is described in figure legends as well as in the list of Source Data Files (pages 42-43).

R scripts used for analyses of flow cytometry and pyrosequencing data are available in Supplementary File 3. Input Files necessary to run the scripts are available in Supplementary File 4. Matlab code used for modeling population growth is available in Supplementary File 5.

Source data produced by flow cytometry (.fcs files) are available at the FlowRepository (<https://flowrepository.org/>) under experiment ID FR-FCM-ZY8Y, FR-FCM-ZYJN, FR-FCM-ZY7E, FR-FCM-ZYJX. Repository IDs are described in the Methods section. Microscopy images used to quantify cell division rates are available on Zenodo (<https://zenodo.org/>) with DOI 10.5281/zenodo.1327545.
